# A novel anti-platelet aggregation target of chinensinaphthol methyl ether and neojusticin B obtained from *Rostellularia procumbens* (L.) Nees

**DOI:** 10.1080/14756366.2019.1609468

**Published:** 2019-05-10

**Authors:** Songtao Wu, Yanfang Yang, Bo Liu, Zhoutao Xie, Weichen Xiong, Pengfei Hao, Wenping Xiao, Yuan Sun, Zhongzhu Ai, Hezhen Wu

**Affiliations:** a Faculty of Pharmacy, Hubei University of Chinese Medicine, Wuhan, China;; b Key Laboratory of Traditional Chinese Medicine Resources and Chemistry of Hubei Province, Wuhan, China;; c Collaborative Innovation Center of Traditional Chinese Medicine of New Products for Geriatrics Hubei Province, Wuhan, China

**Keywords:** Chinensinaphthol methyl ether, neojusticin B, integrin α_IIb_*β*_3_, platelet aggregation, gene chip, network pharmacology, Prometheus NT.48, microscale thermophoresis

## Abstract

This study explored the possible bioactive ingredients and target protein of *Rostellularia procumbens* (L.) Nees. The results of optical turbidimetry revealed that the ethyl acetate extraction obtained from *R. procumbens* (L.) Nees could inhibit platelet aggregation. Gene chip was used to investigate differentially expressed genes. According to the results of the gene chip, the targets of compounds isolated from the ethyl acetate extraction were predicted by network pharmacology. Computational studies revealed that chinensinaphthol methyl ether and neojusticin B may target the integrin α_IIb_β_3_ protein. The results of Prometheus NT.48 and microscale thermophoresis suggested that the molecular interactions between the two compounds with purified integrin α_IIb_β_3_ protein in the optimal test conditions were coherent with the docking results. To our best knowledge, this is the first report to state that chinensinaphthol methyl ether and neojusticin B target the integrin α_IIb_β_3_ protein.

## Introduction

Integrin α_IIb_β_3_ is a major platelet-surface receptor for the regulation of platelet aggregation and thrombosis. The resting integrin α_IIb_β_3_ is curved, and after the platelets are activated and deformed, the integrin α_IIb_β_3_ on the membrane becomes concentrated on the pseudopods of the dendritic platelets and becomes erect. In this process, the third subunit moves in the direction of the cell’s interior, opening the angle set up with the other subunit, such that the fibrin head can fit in and complete the key process of platelet aggregation^1^^,^^2^.

Fibrinogen is attached to the integrin α_IIb_β_3_ protein of one platelet and is linked to the integrin α_IIb_β_3_ protein of another platelet. At the same time, the platelets are grouped together to cause a platelet aggregation cascade. Therefore, integrin α_IIb_β_3_ is a key protein for platelet aggregation^3–5^.

*Rostellularia procumbens* (L.) Nees is mainly obtained from the *Acanthaceae* plants. It is widely distributed in the Taiwan Province and the southwest provinces and has been proven to have a huge potential for the development of Chinese medicine owing to its plant resources, chemical constituents, pharmacological action, and clinical application. It has complex chemical composition^6–8^.

In addition, reports have shown that *R. procumbens* (L.) Nees has significant pharmacological properties such as anti-viral and anti-tumoral^9^^,^^10^. It has also been noted that aqueous extracts of *R. procumbens* (L.) Nees decrease platelet aggregation^11^. Using optical turbidimetry analysis, we were able to identify the active extract. Then, gene chip was used to investigate differentially expressed genes. The targets of isolated compounds were predicted according to the reverse pharmacophore matching model. The platelet aggregation-related genes were found in databases, and antiplatelet aggregation-related gene targets were selected through comparison. The functions of target genes and related pathways were analysed and screened using the DAVID database, and the network of antiplatelet aggregation effect of isolated compounds was constructed using Cytoscape software. Currently, computer-docking analysis is a globally recognized method employed to interpret the relationship between bioactive ingredients and their target protein. Molecular docking is carried out to predict the interaction between compounds and proteins^12–15^. Prometheus NT.48 is used to detect protein stability and screen buffer^16^. Then, we compared the two models of MST and used NT.115 to verify the interaction between the compound and the integrin α_IIb_β_3_ protein^17–26^.

In this study, according to the results of gene chip, the active ingredients of *R. procumbens* (L.) Nees and their target were screened by network pharmacology and dock. The interaction between compounds and the membrane protein integrin α_IIb_β_3_ was verified by MST. It would lay the groundwork for understanding the molecular mechanism involved in the inhibition of platelet aggregation by *R. procumbens* (L) Nees.

## Materials and methods

### Chemical and materials

About 80% ethanol extract, ethyl acetate extract, *n*-butanol extract, water extract, neojusticin B, chinensinaphthol methyl ether, justicidin E, justicidin B, and cilinaphthalide B were obtained from the Key Laboratory for Traditional Chinese Medicine Resources and Chemistry of Hubei Province^27–29^. Deionized water was made available in-lab using a Milli-Q purification instrument (Millipore, Bedford, MA, USA). 5-hydroxytryptophan was obtained from the National Institutes for Food and Drug Control (China). Thrombin was purchased from BioMed Lublin, Poland. AA, bovine serum albumin (BSA), adrenaline, adenosine diphosphate (ADP), PAF, and dimethyl sulfoxide (DMSO) were purchased from Sigma (St. Louis, MO, USA). Analytical grade (purity ≥ 99.9) petroleum ether, ethyl acetate, *n*-butanol, acetone, chloroform, ethanol, and methanol were supplied by Sinopharm Chemical Reagent Co., Ltd (Shanghai, China). RNeasy Mini Kit (QIAGEN, German), RNase-free DNase I (QIAGEN, Düsseldorf, Germany). Pico Reagent Kit (Affymetrix, Santa Clara, CA, USA), GeneChip90 Hybridization, Wash, and Stain Kit (Affymetrix).

### Experimental animals

SD male rats (190–230 g) were obtained from the Hubei Provincial Center for Disease Control and Prevention (Wuhan, China). After feeding for 3 days, we took 5–8 ml of blood from the femoral artery. All experimental procedures were approved by Animal Care and Use Committee of Institute of Materia Medica, People’s Republic of China.

### Screening of active extract inhibiting platelet aggregation

The dry extract of 80% ethanol (0.2172 g), ethyl acetate (0.2172 g), *n*-butanol (0.1080), and water (0.4352 g) were separately placed in a 1 ml measuring bottle. DMSO was precisely added into the 1 ml measuring bottle to weigh the sample. Ultrasonic treatment (power 120 W, frequency 40 kHz) was used to dissolve the sample. DMSO was used to make up the lost weight to obtain the sample storage liquid.

Platelet-rich plasma (PRP) was prepared by centrifugation of fresh blood at 200*g* for 10 min at room temperature and aspirating the supernatant. Platelet-poor plasma was then sedimented by centrifugation of residual blood at 800*g* for 10 min at room temperature. Blood platelet aggregation was monitored by platelet turbidity, with 0% aggregation calibrated as the absorbance of platelet-poor plasma and 100% aggregation as the absorbance of PRP. PRP was incubated with the drug at 37 °C for 20 min and then stimulated with ADP. The aggregation of PRP (pre-incubated with the tested plant fraction) in response to 10 µM ADP was recorded using an aggregometer (LBY-NJ4).

### Extraction and detection of total RNA in two groups of platelets

The platelet of the control group and ethyl acetate group were centrifuged at 4 °C and 12,000 rpm for 10 min. Total RNA was isolated with an RNeasy Mini Kit. The RNA concentration and A260/A280 ratio were determined using an SMA 3000 microspectrophotometer (Meriton, Beijing, China). The results revealed that the ratio was 2.00:2.05, which indicated high purity of the extracted RNA, which was deemed suitable for subsequent analysis. Following this, a total of 1 µg RNA was used for 1% agarose gel electrophoresis. The ratio of 28S/18S was then determined using a JS-380A Bioanalyzer (Shanghai Culture and Technology Co., Ltd., Beijing, China) to determine the quality of RNA. If the RNA integrity number (RIN) ≥ 7.0 and 28S/18S > 0.7, samples were transcribed by a Pico Reagent Kit. Following this, samples were prepared using a GeneChip90 Hybridization, Wash, and Stain Kit (Affymetrix, Thermo Fisher Scientific, Inc., Santa Clara, CA, USA). Then, chips were scanned using a GeneChip Scanner 3000 (Affymetrix, Thermo Fisher Scientific, Inc.).

### Prediction and analysis of differentially expressed target genes

Based on the results of the gene chip, we conducted a network pharmacology study. The PharmMapper Server (http://lilab.ecust.edu.cn/pharmmapper/index.php) is a freely accessed web server used to identify potential target candidates for given probe isolated compounds using a pharmacophore mapping approach. And GeneCards (http://www.malacards.org/) and MalaCards (https://www.genecards.org/) were used for potential target screening from the results of gene chip. All potential target genes were synthesized and uploaded to the DAVID (https://david.ncifcrf.gov/summary.jsp) database. Following this, the functions and signalling pathways of target genes, as well as pathway enrichment, were investigated via Gene Ontology (GO; http://geneontology.org/) and Kyoto Encyclopedia of Genes and Genomes (KEGG) pathway analyses (http://www.genome.jp/kegg/ko.html).

### Construction of the network

The isolated compounds and target genes were imported into Cytoscape 3.6.1 software (CA, USA) to build an active compound/target gene/pathway network and a target gene/platelet aggregation-pathway network. The isolated compounds and target genes were input as the node. If there was a connection between two nodes, the edge was used to show the connection.

The network was then analysed with the network analyse function. High-degree gene targets in the protein interaction network were analysed. According to the results of KEGG enrichment, the pathways with higher counts were selected to analyse their key targets. Meanwhile, the genes suitable for the analysis of the targets were obtained through comparative analysis from the literature and database.

### Docking trail

The virtual dock was performed against integrin α_IIb_β_3_ using the AutoDock software to study the interaction between the isolated compounds and integrin α_IIb_β_3_ protein. The 3D ligand file was prepared using PubChem. The three-dimensional structure of the integrin α_IIb_β_3_ protein was retrieved from Protein Data Bank (PDB:2VC2). Before the docking trial, nonpolar hydrogen atoms were merged, nonintegral charges on ligand file and integrin α_IIb_β_3_ were corrected, and Gasteiger charges were also added by using AutoDock tools. Torsion bonds of the compounds were selected and defined. The three-dimensional grid box was created by using an AutoGrid algorithm (part of the AutoDock package) to evaluate the binding energy on the macromolecule coordinates. The grid maps representing the intact ligand in the actual docking target site were also calculated with AutoGrid. The three-dimensional grid box with 60 Å grid size (*x*, *y*, *z*) with a spacing of 0.300 Å was created. Eventually, cubic grids encompassed the binding site where the intact ligand was embedded. Finally, AutoDock was used to calculate the binding free energy of a given compound’s conformation in the macromolecular structure, while the probable structural inaccuracies were ignored in the calculations.

### Protein formulation screen

To identify optimal testing and storage conditions for integrin α_IIb_β_3_, the protein was subjected to a thermal unfolding formulation screen. The Prometheus NT.48 measured the thermal unfolding profiles of proteins by detecting even minute changes in the emission properties of the amino acid tryptophan upon unfolding. Five different buffer conditions were tested to address the thermal unfolding transition temperatures of the antibody in dependence of detergent and buffer substances. The buffer screen comprised the following buffer substances and detergent combinations: Tris–tween, Tris–CHAPS, Tris–DDM, Hepes–DDM, Hepes–CHAPS, Hepes–tween, PBS–DDM, PBS–CHAPS, and PBS–tween, each at 20 mM and 0.1% final concentration, each in the presence of 100 mM NaCl and 1 mM CaCl_2_. Thermal unfolding experiments were carried out on the Prometheus NT.48 at a heating rate of 1 °C/min. The measurement of the F350/F330 of 48 capillaries at a given temperature was performed in 90 min.

### NT.LabelFree analysis

As integrin α_IIb_β_3_ is a membrane protein, we used NT.LabelFree to prevent the label from affecting the results. Titration series with ligand concentrations varying between 0 and 1000 mM were prepared in the Prometheus NT.48 optimized buffer. SD test was required before measurement.

Approximately, 3 µl was loaded into NT.LabelFree standard-treated capillaries (Nanotemper). MST experiments were performed at 40% MST (infra-red laser) power and 60% LED power at 25 °C using the Monolith NT.LabelFree Instrument (Nanotemper, Munich, Germany). Ratios between normalized initial fluorescence and after temperature-jump and thermophoresis were calculated and averaged from 5 to 9 independent runs. Means of fluorescence intensity obtained by the MST measurements were fitted, and the resultant *K*_d_ values were given together with an error estimation from the fit by the built-in formula of the analysis software.

### NT.115 analysis

The fluorescence of the ligand interfered with the result. This was further exacerbated when using label-free thermophoresis owing to the additional noise present in measuring fluorescence in ranges where inherent fluorescence of the protein itself is measured. After the SD test experiment verified that the label had little effect on the protein, we used NT.115 for measurement.

All the compounds were analysed with the concentration gradient of 50 µM with 20 µM of CviR that was labelled Monolith NTTM Protein Labeling Kit RED–NHS (Cat Nr: L001) before instrumental analysis. LED power was 20% and Prometheus NT.48 optimized buffer was used only for the analysis.

Analysis was performed on Monolith Nano Temper (NT)115 and its accessory, i.e. standard-treated 4 µL volume glass capillaries were employed to measure the molecular interaction (Nano Temper Technologies GmbH, Munich, Germany). Means of the fluorescence intensity obtained by the MST measurements were fitted, and the resultant *K*_d_ values were given together with an error estimation from the fit by the built-in formula of NT1.5.41 analysis software (Nanotemper, Munich, Germany).

## Results

### Anti-aggregant effect of the extract

**Figure 1.**
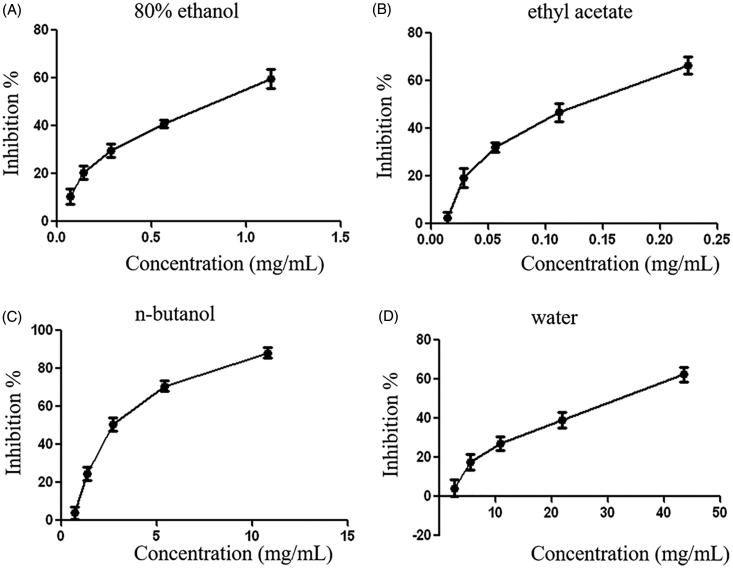
Curves of inhibition of platelet aggregation induced by ADP of (A) 80% ethanol, (B) ethyl acetate, (C) *n*-butanol, and (D) water extract from *Rostellularia procumbens* (L.) Nees.

After preincubation with the extract for 20 min, platelets were stimulated by ADP (10 μM). Figure 1 shows that the different extract concentrations inhibit platelet aggregation induced *in vitro* by ADP. IC50s were 0.7761 mg/ml (80% ethanol), 0.1202 mg/ml (ethyl acetate), 2.863 mg/ml (*n*-butanol), and 29.06 mg/ml (water) (*n* = 3). Regarding the extract, stronger inhibition of aggregation was observed with ethyl acetate; others were relatively poor at the same concentration. The remaining extract showed different degrees of anti-aggregation.

### Microarray results

Compared with the control group, hundreds of genes were revealed to be differentially expressed in platelet in the EtOAc extract group (Table 1). In EtOAc extract group, PLCB2, PRKCA, GNAQ, MAPK10, MAPK8, MAPK11, MAPK14, GNAI2, PIK3CG, and PIK3R1 were markedly underexpressed in the liver tissues of the model group compared with the control group.

**Table 1.** The top different 42 genes of platelet expression between the ethyl acetate extract group and blank group.

Gene symbol	Betweenness	Indegree	Outdegree	Degree
PRKACG	94,737.81	6	44	50
ADCY5	75,328.3	32	18	50
PRKCA	66,572.86	11	32	43
CREBBP	66,251.38	15	26	41
PTGS2	53,449.43	19	11	30
PLCB2	44,137.16	35	26	61
PLCB4	44,137.16	35	26	61
NFKB1	43,688.56	18	29	47
TJP1	41,733.01	25	19	44
CTNNB1	37,713.52	24	8	32
ENPP3	34,186.49	14	14	28
ADCY4	29,712.44	30	18	48
GLB1	28,178.83	10	10	20
GJA1	26,950.54	11	9	20
ACTB	25,842.48	30	16	46
CYP2C9	23,028.48	28	28	56
CDC42	21,745.7	11	10	21
MAPK10	21,496.44	28	17	45
MAPK8	21,496.44	28	17	45
CYP4A11	21,389.71	20	19	39
PIK3CG	21,374.9	48	14	62
NT5E	21,374	22	22	44
PTGS1	18,877.33	10	11	21
STAT1	18,844.37	14	13	27
PIK3R1	18,754.65	43	15	58
PLD2	18,491.23	14	14	28
PIK3CD	18,266.94	42	14	56
MAOB	18,000.65	13	13	26
GSK3B	17,754.69	6	13	19
MAPK11	17,135.02	24	21	45
MAPK14	17,135.02	24	21	45
EGFR	17,081.33	21	18	39
VEGFA	16,074.26	11	6	17
ATF4	15,173.42	16	8	24
CRKL	14,861.02	20	10	30
PPP2CB	14,831.02	6	10	16
PPP2R1A	14,831.02	6	10	16
GBA	14,292.23	7	7	14
IMPAD1	13,152.14	9	9	18
HK3	13,138.31	13	12	25
CYP3A4	12,935.99	24	24	48
ITGB2	12,473.6	5	12	17

### Prediction and analysis of the target genes

**Figure 2.**
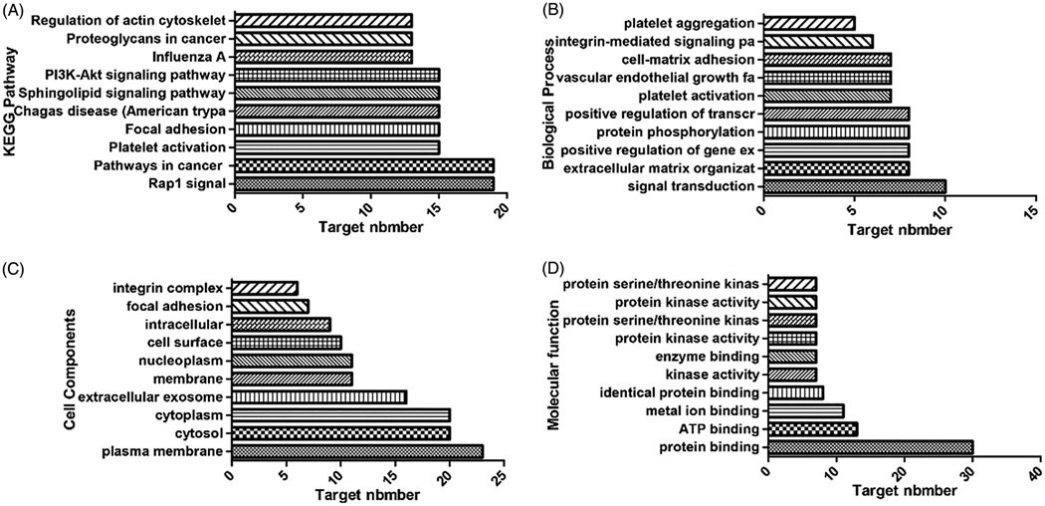
Top 10 components of the KEGG pathway and GO enrichment analyses. (A) KEGG pathways. (B) Biological process. (C) Cell components. (D) Molecular function.

All potential target genes were synthesized and uploaded to the DAVID database for KEGG pathway annotation and GO enrichment. The threshold was set as *p* ≤ .05, and the pathways or gene functions with higher count were analysed. The top 10 pathways were graphed by GraphPad Prism 6 (Figure 2).

KEGG pathway annotation showed that 35 of the 35 potential target genes were enriched (100%) and involved 137 pathways, and 110 of these pathways were significantly correlated with the target genes (*p* ≤ .05). The following pathways had the largest number of genes involved: Rap1 signalling pathway (19, 54.3%), pathways in cancer (19, 54.3%), platelet activation (15, 42.9%), focal adhesion (15, 42.9%), Chagas disease (American trypanosomiasis) (15, 42.9%), and sphingolipid signalling pathway (15, 42.9%). GO enrichment analysis showed that the number of genes involved in the CC, MF, and BP targets was 35 (100%). CC enrichment was mainly involved in following target genes: plasma membrane (23, 65.7%), cytosol (20, 57.1%), and integrin complex (6, 17.1%). MF enrichment was mainly involved in the following target genes: protein binding (30, 85.7%), ATP binding (13, 37.1%), and metal ion binding (11, 31.4%). BP enrichment was mainly involved in the following target genes: signal transduction (10, 28.6%), platelet activation (7, 20%), and platelet aggregation (5, 14.3%).

### Construction of the network

**Figure 3.**
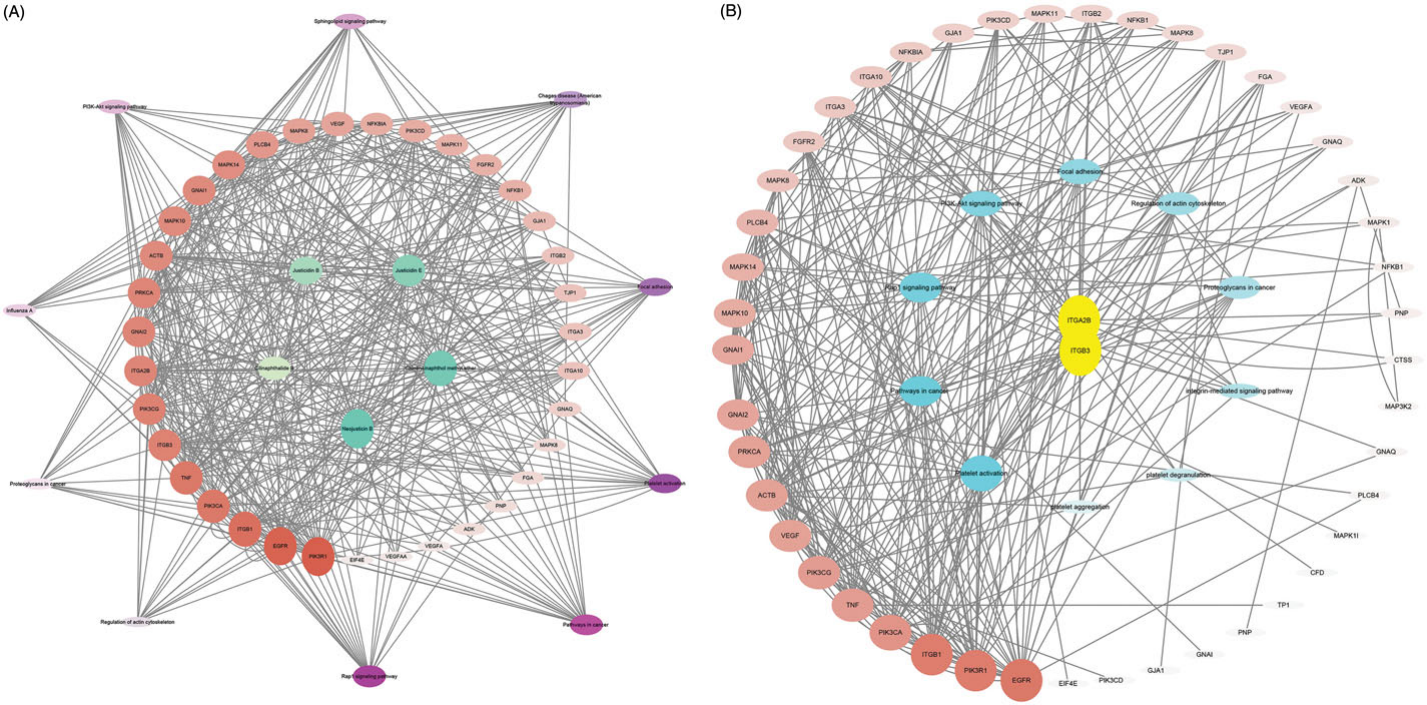
Network diagram constructed by Cytoscape. (A) Network diagram of active components/target genes/enrichment pathways. (B) Network diagram of target gene/platelet aggregation-related pathways.

According to the results of gene chip, target genes in the top 10 pathways and compounds were selected to construct an active compound/target gene/pathway network and a target gene/platelet aggregation-related pathway network. (Figure 3). The network diagram shows the synergistic effect of various compounds on multiple targets when *R. procumbens* (L.) Nees plays a role in antiplatelet aggregation effects.

The network analyse tool was used to analyse the network, and genes with higher degree were associated with more genes. It can be considered that the corresponding protein of the genes plays an important role in central correlation when *R. procumbens* (L.) Nees plays an antiplatelet aggregation role. The integrin α_IIb_β3 had the highest degree, as it was the intersection of all platelet-related pathways.

The results were compared with those of the KEGG pathway analysis and combined with the literature study. Integrin α_IIb_β_3_ was selected as the target for the binding validation experiment.

### Molecular interaction of inhibitors against integrin α_IIb_β_3_

The virtual dock was performed against integrin α_IIb_β_3_ by using the AutoDock software to study the interaction between the isolated compounds and integrin α_IIb_β_3_ protein. The native ligand (MERCK) could form three H-bonds with ARG214, ASN215, SER123, and 2 π–π stacking with PHE160 and TYR190, which was used as a reference value and pattern of interaction for the pose analysis. Neojusticin B, chinensinaphthol methyl ether, justicidin E, justicidin B, and cilinaphthalide B showed binding free energy of −5.875, −5.130, −4.707, −4.471, and −4.575 (Table 2). Neojusticin B was in a position to form 2 H-bonds with amino acids ARG214 and ALA218 along with a π–π stacking with PHE160. Chinensinaphthol methyl ether could form 2 π–π stacking with PHE160 and a pi-cation with MG1460. Whereas, justicidin E could only form one pi-cation with ARG214 and two π–π interactions with TYR190 and PHE160. In contrast, justicidin B and cilinaphthalide B could only form 1 π–π stacking with PHE160. After pose analysis, based on the binding free energy and H-bond forming ability, the ligands were chosen for further studies. PHE160, ARG214, ALA218, and MG1460 were considered to be the active site residues. The main difference in the structure of the five compounds is mainly the methoxy group on the parent phenyl ring and the five-membered ring containing two oxygen molecules in the structural formula. It is inferred that they are key groups in the interaction of compounds with proteins.

**Table 2.** AutoDock binding free energies (ΔGb) and bonds of the docked inhibitors against integrin α_IIb_β_3_.

			Bonds between groups of compounds and amino acids of integrin α_IIb_β_3_		
PDB code	Inhibitors	ΔGb (kcal/mol)	Groups of comp.	Amino acid	Bonds name
	Neojusticin B	−5.875	O	ARG214	H-bond
			O	ALA218	H-bond
			Benzene ring	PHE160	π–π stacking
	Chinensinaphthol methyl ether	−5.130	Benzene ring	MG1460	Pi-cation
			Benzene ring	PHE160	π–π stacking
2VC2			Five-membered ring	PHE160	π–π stacking
	Justicidin E	−4.707	Five-membered ring	PHE160	ππ–π stacking
			Five-membered ring	TYR190	π–π stacking
			Five-membered ring	ARG214	Pi-cation
	Justicidin B	−4.471	Benzene ring	PHE160	π–π stacking
	Cilinaphthalide B	−4.575	Benzene ring	PHE160	π–π stacking

### Optimal test conditions

**Figure 4.**
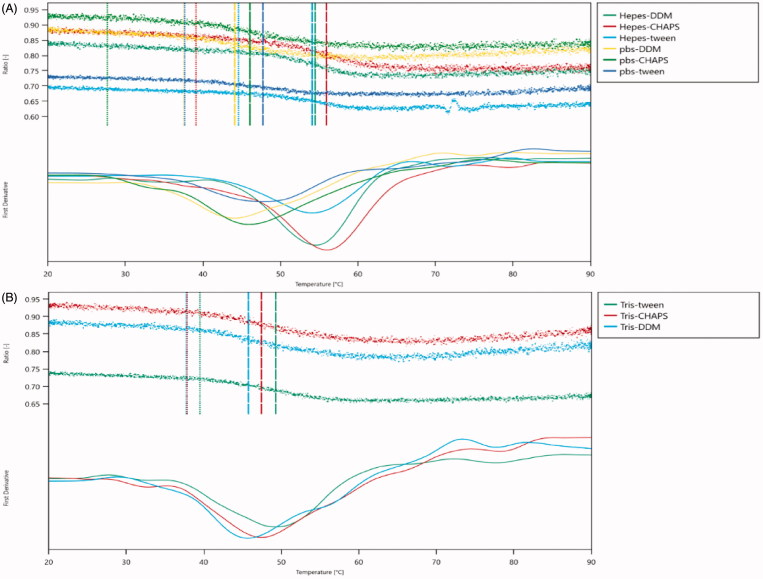
Thermal unfolding curves. (A) Thermal unfolding curves in the presence of Hepes and pbs. (B) Thermal unfolding curves in the presence of Ttis. Insets show the detergent-dependence of the first unfolding transition midpoint (Tm1).

A plot of the unfolding transition temperature versus respective buffer substance and detergent showed that the thermal stability strongly depended on the detergent (Figure 4). The protein displayed constant thermal stabilities at a temperature range from 20 °C to 30 °C. Moreover, a strong decrease in unfolding transition temperatures of different buffers without Hepes was observed, pointing towards a significant destabilization under these conditions. Conversely, the onset for the ratio of the buffer with Hepes concentration is around 40 °C, especially the Hepes–DDM. These results show that the optimized buffer (0.1% DDM, 20 mM Hepes, 100 mM NaCl, 10 mM CaCl_2_) delivers the highest quality thermal unfolding data for integrin α_IIb_β_3_ buffer-screening campaigns.

### NT.LabelFree

**Figure 5.**
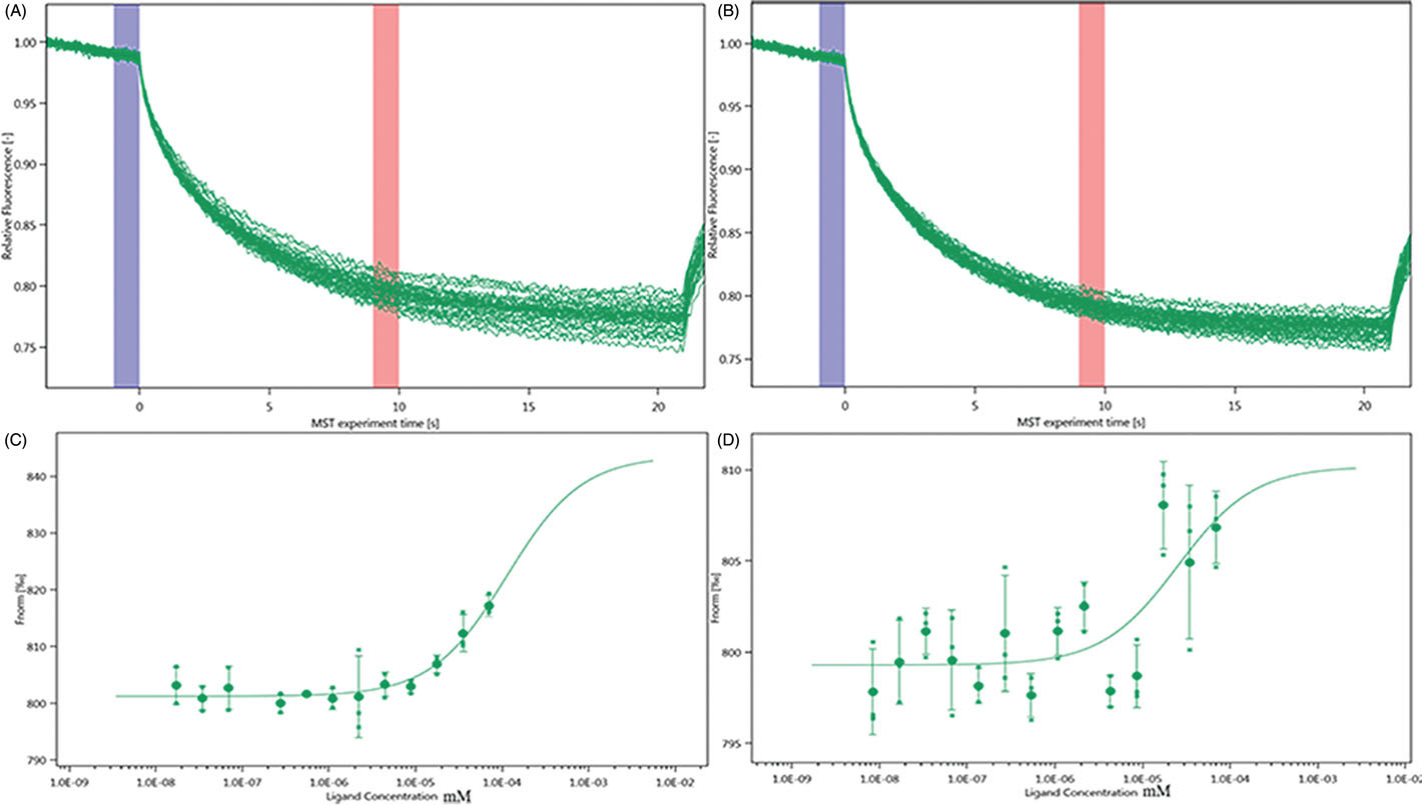
Molecular interaction of integrin α_IIb_β_3_ using NT.LabelFree analysis. (A) MST time traces of 16 different neojusticin B concentrations (ranging from 0.0173 to 71 mM). (B) MST time traces of 16 different chinensinaphthol methyl ether concentrations (ranging from 0.00845 to 69.3 mM). (C) Dependence of the MST signal on the neojusticin B concentration (measured 30 s after turning on heating; data from A). The solid line is a fit with Michaelis–Menten kinetics, yielding an apparent dissociation constant of *K*_d_ = 113.47 ± 76.536 nm. (D) Dependence of the MST signal on the chinensinaphthol methyl ether concentration (measured 30 s after turning on heating; data from B). The solid line is a fit with Michaelis–Menten kinetics, yielding an apparent dissociation constant of *K*_d_ = 25.22 ± 34.934 nm.

MST experiments were performed to detect the molecular interaction between inhibitors and integrin α_IIb_β_3_. Owing to the influence of ligand fluorescence, we obtained unsatisfactory results under the SD test pass condition, accompanied by relatively large errors. Then, the dissociation constant (*K*_d_) was calculated. The *K*_d_ values of neojusticin B and chinensinaphthol methyl ether were 113.47 ± 76.536 nm and 25.22 ± 34.934 nm, respectively (Figure 5).

### NT.115

**Figure 6.**
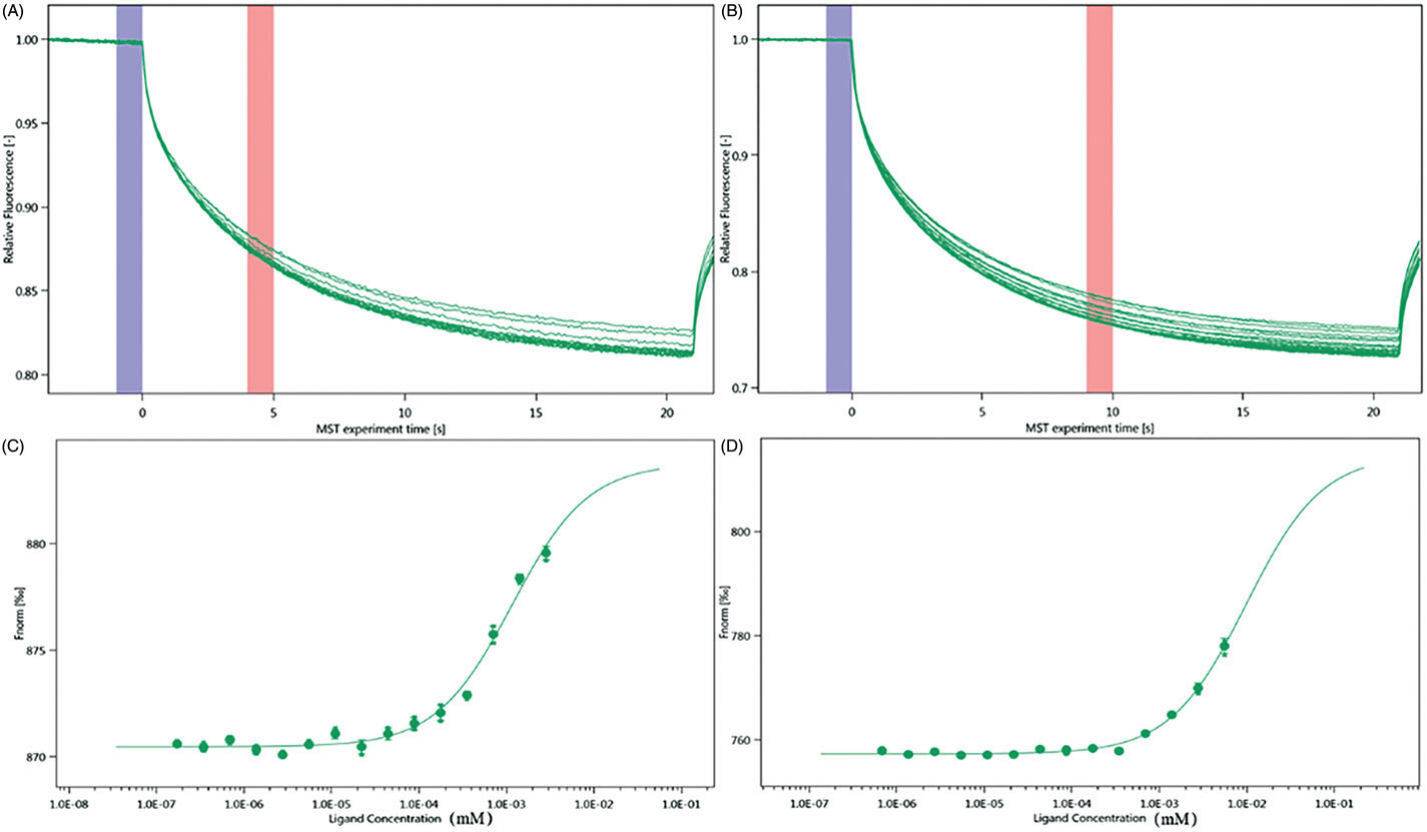
Molecular interaction of integrin α_IIb_β_3_ using NT.115 analysis. (A) MST time traces of 16 different neojusticin B concentrations (ranging from 0.000173 to 2.84 mM). (B) MST time traces of 16 different chinensinaphthol methyl ether concentrations (ranging from 0.000677 to 5.55 mM). (C) Dependence of the MST signal on the neojusticin B concentration (measured 30 s after turning on heating; data from A). The solid line is a fit with Michaelis–Menten kinetics, yielding an apparent dissociation constant of *K*_d_ = 1.1592 ± 2.5447 μm. (D) Dependence of the MST signal on the chinensinaphthol methyl ether concentration (measured 30 s after turning on heating; data from B). The solid line is a fit with Michaelis–Menten kinetics, yielding an apparent dissociation constant of *K*_d_ = 9.8229 ± 0.21873 μm.

The SD test verified that the label had less effect on the protein, and we used NT.115 for the experiment. As differences in normalized fluorescence of the bound and unbound state will allow determination of the fraction bound, the dissociation constant was thus calculated. All values were multiplied by a factor of 1,000, which yielded the relative fluorescence change in per thousand. The *K*_d_ values of neojusticin B and chinensinaphthol methyl ether were 1.1592 ± 2.5447 μm and 9.8229 ± 0.21873 μm (Figure 6).

## Discussion

*Rostellularia procumbens* (L.) Nees has been used in herbal medicines for promoting blood circulation and pain relief. Modern pharmacological studies have shown that *R. procumbens* (L.) Nees has a good anti-platelet aggregation effect^11^. In this study, optical turbidimetry was used to measure the anti-platelet aggregation effects of the extract obtained from *R. procumbens* (L.) Nees. The results indicated that the ethyl acetate extract derived from dried *R. procumbens* (L.) Nees samples significantly reduced platelet aggregation. However, the chemical composition of this plant material is quite complex. Therefore, it was difficult to determine the target protein by using traditional methods.

The results of gene chip illustrated that ethyl acetate extract could inhibit Gq-PLC-PKC pathway and Gi-PI3K-MAPKs pathway. The down-regulation of GNAI1 gene related to the regulation of AC kinase was also observed, indicating that the ethyl acetate site can reduce the inhibitory effect of Gi and enhance the activity of AC kinase. This result indicated that the ethyl acetate site could regulate the GI-AC-CAMP signalling pathway. Above all, ethyl acetate might inhibit platelet aggregation by inhibiting Gq-PLC-PKC, Gi-PI3K-MAPKs and other signalling pathways. Based on the results of the gene chip, the targets of isolated compounds were predicted according to the reverse pharmacophore matching model. The platelet aggregation-related genes were found in databases, and antiplatelet aggregation-related gene targets were selected through comparison. GO enrichment analysis found that the targets involved plasma membrane, cytosol, extracellular exosome, integrin complex, and other cell compartments. At the molecular level, the targets were involved in protein binding, ATP binding, enzyme binding, and other molecular activities, and they were related to platelet aggregation, platelet activation, and platelet degranulation. It indicated that the *R. procumbens* (L.) Nees may inhibit platelet aggregation by binding to membrane proteins, affecting its energy utilization and activation, etc. The pathway enrichment results also indicated that *R. procumbens* (L.) Nees may play an antiplatelet aggregation role by inhibiting the key targets of the platelet activation signalling pathway, such as integrin α_IIb_β_3_, it was the intersection of all platelet-related pathways. Docking calculations were performed using the AutoDock protocol to discover the active ingredients of *R. procumbens* (L.) Nees responsible for the mechanism involved in the inhibition of platelet aggregation. The results showed that neojusticin B and other compounds could interact well with the integrin α_IIb_β_3_ protein. Then, the active site was copied from integrin α_IIb_β_3_ (PDB code: 2vc2). Finally, results from the docking analysis proved that these compounds could interact with integrin α_IIb_β_3_ protein with good docking score. These compounds attracted the integrin α_IIb_β_3_ protein in platelets through specific residual interaction with PHE160, ARG214, and ALA218 of integrin α_IIb_β_3_. In addition, there were also two π–π interactions with TYR190 and MG1460 with integrin α_IIb_β_3_ side chains. Our results speculate that these compounds can inhibit the activity of integrin α_IIb_β_3_ in inhibiting platelet aggregation by the methoxy group on the parent phenyl ring and the five-membered ring containing two oxygen molecules in the structural formula.

It is well known that membrane proteins are less stable, and this has shown to have a massive impact on the MST (NT.LabelFree) experiment^27^. To identify optimal test and storage conditions for the membrane protein integrin α_IIb_β_3_, the protein was subjected to a thermal unfolding formulation screen of the Prometheus NT.48. NanoTemper’s on-the-fly technology allows to measure 48 samples in parallel providing more than 10 data points per minute. Formulation developments benefit from the ultra-high resolution that are not compromised by aggregation, but at the same time, offer ease of use. Prometheus NT.48 results suggest that Hepes is the best detergent. The onset for the ratio of Hepes-DDM is 46.1 °C, which is higher than other buffers. This indicates that the protein is stable even at 46.1 °C at this condition. Thus, we chose this buffer for the following MST experiments. This attempt was not reported before.

MST is a powerful technique to measure biomolecular interactions that are based on thermophoresis—the movement of molecules in a temperature gradient. This technique was reported to be highly sensitive such that it allows precise quantification of molecular interactions^28^. Because of the influence of ligand fluorescence, we obtained unsatisfactory results, accompanied by relatively large errors. The results of the Prometheus NT.48 experiment showed that protein stability was relatively good, and the labelled protein SD test was qualified; hence, we experimented further with NT.115 and obtained better results. MST results suggest that two selected compounds have potential molecular interaction with integrin α_IIb_β_3_ (Figure 5). The *K*_d_ values of neojusticin B and chinensinaphthol methyl ether were 1.1592 ± 2.5447 μm and 9.8229 ± 0.21873 μm. These data suggest that neojusticin B and chinensinaphthol methyl ether have a similar interaction pattern to that of MERCK, and show good interaction with integrin α_IIb_β_3_. According to Seidel et al.^29^, the fitting curve may be either S-shaped or mirror S-shaped. The standard symbol of MST amplitude (change in normalized fluorescence) depends on the chemistry of the compound that is titrated, its binding site, and the conformational change induced upon binding. Neojusticin B and chinensinaphthol methyl ether show a positive slope suggesting a strong conformational change induced upon complex formation. Probably their π–π interactions play a major role in conformational change. We speculate that they might interfere with the platelet aggregation mechanism by negatively influencing the conformational changes required for the integrin α_IIb_β_3_ activation.

In addition, previous animal experiments showed that the LD_50_ of ethyl acetate extract was 2419.4 mg/kg, meeting the standard of low toxic. The results showed that the toxicity of ethyl acetate extract was low and its clinical use was safe. Then, when the ethyl acetate extract was orally administered, 23.6 mg/kg could significantly extend the clotting time. The serum pharmacochemistry assay also proved that the active components had high bioavailability. Combined with the study of target protein in this article, it lays a foundation for the development of new drugs in the future^33^.

## Conclusion

In this study, our results demonstrate that the ethyl acetate extract may play an anti-platelet aggregation role through integrin α_IIb_β_3_, and the compounds neojusticin B and chinensinaphthol methyl ether are targeted to it. We believe our findings would provide a better foundation for further understanding of the mechanism of *R. procumbens* (L.) Nees intervention in platelet aggregation.
